# Temporal Synchrony Detection and Associations with Language in Young Children with ASD

**DOI:** 10.1155/2014/678346

**Published:** 2014-12-29

**Authors:** Elena Patten, Linda R. Watson, Grace T. Baranek

**Affiliations:** ^1^Department of Audiology and Speech Pathology, University of Tennessee Health Science Center, 434 South Stadium Hall, Knoxville, TN 37996, USA; ^2^Division of Speech & Hearing Sciences, CB No. 7190, University of North Carolina at Chapel Hill, Chapel Hill, NC 27599, USA; ^3^Division of Occupational Sciences, CB No. 7122, University of North Carolina at Chapel Hill, Chapel Hill, NC 27599, USA

## Abstract

Temporally synchronous audio-visual stimuli serve to recruit attention and enhance learning, including language learning in infants. Although few studies have examined this effect on children with autism, it appears that the ability to detect temporal synchrony between auditory and visual stimuli may be impaired, particularly given social-linguistic stimuli delivered via oral movement and spoken language pairings. However, children with autism can detect audio-visual synchrony given nonsocial stimuli (objects dropping and their corresponding sounds). We tested whether preschool children with autism could detect audio-visual synchrony given video recordings of linguistic stimuli paired with movement of related toys in the absence of faces. As a group, children with autism demonstrated the ability to detect audio-visual synchrony. Further, the amount of time they attended to the synchronous condition was positively correlated with receptive language. Findings suggest that object manipulations may enhance multisensory processing in linguistic contexts. Moreover, associations between synchrony detection and language development suggest that better processing of multisensory stimuli may guide and direct attention to communicative events thus enhancing linguistic development.

## 1. Introduction

Communication impairment is a hallmark feature of autism spectrum disorder (ASD) [1]. Identifying latent behaviors necessary for communication to develop normally could provide early diagnostic and prognostic indicators, suggest mechanisms underlying impairments, and inform the development of novel habilitative interventions. The ability to detect and benefit from multisensory auditory-visual stimulation early in development may be one of the latent prelinguistic behaviors which is critical for communication to develop normally; in autism, early impairments in multisensory processing could place the developing child on a trajectory that yields increasingly abnormal attention and communication.

Bahrick and Lickliter's [2, 3]* Intersensory Redundancy Hypothesis* proposes that an infant's ability to detect intersensory redundancy (i.e., stimulation across senses from a unitary event) guides perceptual development such that global amodal properties (i.e., not specific to a single sensory modality, such as periodicity) are processed before local unisensory details. Auditory-visual temporal synchrony, for example, is a form of intersensory redundancy and is a condition of stimulation that leads to the binding of multisensory information. This phenomenon allows properties of an event to “pop out,” allowing further processing of the unitary event while preventing incorrect binding of either auditory or visual stimuli with unrelated adjacent stimuli. For example, attention is directed at the temporally synchronous presentation of a mouth moving and the speech of a person in a crowd while other auditory and visual stimuli fade into the background. In fact, by two months of age, infants are able to match speech and lip movement pairings [4]. This facilitating effect of temporal synchrony on attention is present in infancy and engages infants in important communication and social events, providing fertile ground for language to develop [2, 3].

Infant directed speech is heavily laden with auditory and visual cues that draw attention to the most salient aspects of the communication stream and includes synchronized and exaggerated voice and facial expression (for review, see Ratner, 2013 [5]). Moreover, there is evidence that caregivers consciously or subconsciously take advantage of temporal synchrony during interactions. Smith and Strader (2014), for example, found that caregivers temporally align their voice and head movements when communicating with their infants [6].

Research on children with ASD, on the other hand, suggests that they do not show the same degree of preference for infant directed speech as do typically developing children [7]. Thus, the benefits of intersensory redundancy may be reduced in children with ASD in that their attention may not be drawn to the most important aspects of communication. Evidence that this may be the case comes from research suggesting that children with ASD are more likely to incorrectly link a spoken label with an object [8].

Intersensory redundancy has been studied in several different contexts, both linguistic and nonlinguistic, as well as natural and artificial. Linguistic stimuli use speech sounds (phonemes, true words, or speech streams), whereas nonlinguistic stimuli are environmental sounds such as one object striking another. The sight of an object accompanied by the sound it makes (e.g., keys dropping) creates intersensory redundancy as does the sight of moving lips accompanied by speech sounds. These examples are natural and draw attention; thus, the observer is repeatedly exposed to intersensory invariance (i.e., stable, predictable patterns or regularities). Since children prefer intersensory redundancy and since linguistic patterns tend by nature to be intersensorily redundant, they may be a critical form of input during language development and may encourage infants to tune in to the most salient aspects of spoken language [9] (for a broader view of perceptual learning, see Gibson, 1969 [10]).

Intersensory redundancy can also be manufactured such as when an object is moved in synchrony with a verbalization. Unlike natural intersensory redundancy, artificial intersensory redundancy can be manipulated and is potentially useful for teaching. This type of stimulation, sometimes referred to as “multimodal motherese,” is linked to better lexical learning in infants [11]. Further, it is preferred by infants over communication without additional cues [12] and appears to heighten and draw attention to the communicative event [13, 14]. It may be that the temporal redundancy created by the movement of an object paired with the spoken label is what triggers the infant to look at the object, thus encouraging joint attention [9], which is an important precursor to language [15]. In fact, infants engage in more joint attention with their mothers, demonstrate more attention to targets, and show better learning of new words when their mothers use more object-speech temporal synchrony in their communication (i.e., “multimodal motherese”) [11]. Multiple studies have demonstrated improved attention and learning as a result of presenting object-speech temporal synchrony [16–20]. For example, seven-month-olds are able to learn to associate a speech sound with an object only when the auditory and moving visual stimuli are temporally aligned [17] and two-month-olds can detect changes in syllable-object pairings when temporal synchrony is present [19].

Such findings suggest that the ability to detect synchrony may be important for early learning. Rader and Goldring-Zukow (2010) found that moving an object in synchrony with the label drew infants' attention from the speaker's face to the object and, more importantly, was associated with better word learning [21]. It may be that children with ASD can detect synchrony under certain conditions (e.g., artificial versus natural) and glean associated benefits. To date, the effects of infusing artificial intersensory redundancy into language learning in language disabled populations have not been studied. However, a recent study by Gogate et al. (2014) found that preterm infants are less sensitive to multimodal synchrony compared to term infants, which may be causally related to word mapping delays in this group [22]. This finding suggests that early impairments in synchrony detection could negatively impact language development in populations with disabilities.

Difficulty with intersensory processing is well established in autism (for reviews, see [23, 24]); however, the nature of the deficit, particularly the impact on language development, is far less studied. Bahrick and Todd [25] have described ways in which early impairments in detection of intersensory redundancy, including detection of temporal synchrony, may trigger a cascade of disordered developments that yield symptoms of autism. The Intersensory Redundancy Hypothesis describes a hierarchical ordering of attention based on salience of perceptual information. Information that is redundantly specified (e.g., the sound and lip movement of a speaker) is prioritized over nonredundantly specified information (e.g., the color of the speaker's shirt) within the same event. It may be that intersensory redundancy does not cue this prioritized processing in children with ASD. Overall stimulus perception might not be organized from global to local but rather as a disjointed percept, possibly giving the impression of preference for local processing at the expense of global processing (e.g., [26, 27]).

Although there is a fair amount of evidence for impairments in intersensory processing in children with autism, some studies show intact intersensory processing. For example, when given a simple discrimination task such as matching the sound of keys with a visual image of keys, intersensory processing appears to be intact [28]. This may be cognitively mediated, indicating that the child knows the sound of keys regardless of the temporal construction. Findings in children with autism are limited and mixed regarding detection of audio-visual temporal synchrony when the visual stimuli to be matched with the sound are identical with the exception of onset time. Evidence both for and against audio-visual temporal synchrony detection in natural, nonlinguistic contexts has been found. Bebko and colleagues found children with ASD detected audio-visual synchrony in a nonlinguistic, natural context [29], while Bahrick et al. [30] did not. Neither study found evidence that children with autism discriminate between temporally synchronous and asynchronous events in linguistic audio-visual contexts. These studies featuring linguistic stimuli used paradigms that displayed only faces as the visual stimuli [29, 30], such that the temporal synchrony, both natural and linguistic, occurred between the lips moving and the words spoken. This lack of discrimination in face-voice contexts may suggest an underlying impairment in intersensory processing of complex stimuli or, alternatively, perhaps difficulties in face processing negatively impact synchrony discrimination for children with autism (e.g., [31, 32]). Although research demonstrates that typically developing children benefit in terms of attention and language given temporal synchrony between object-speech stimuli independent of the presence of faces and moving lips [16–20], it is unknown whether or not children with autism can detect temporal synchrony between object movement and speech stimuli (artificial, linguistic synchrony). Some of the previously mentioned studies demonstrate that children with ASD can detect temporal synchrony while other studies have found they cannot. If children with ASD demonstrate attention to object-speech synchrony, they may be better positioned to take advantage of lexical training [16–20]. That is, the benefits of synchrony detection may be just powerful with object-speech stimuli as with mouth-speech stimuli, in light of findings by Yu and Smith (2012) who found that typically developing toddlers look mainly at their mother's hand during object naming as opposed to her face [33]. However, even if children with autism are able to detect object-speech temporal synchrony in this context, it would be important to determine if this ability is associated with any developmental skills, such as language, adaptive, or social skills.

We examined children with autism to determine if they could detect temporal synchrony in a linguistic context featuring object movement and verbal labels as described in Gogate et al. [34]. Additionally, we examined the extent to which the amount of time they spent looking toward temporally synchronous audio-visual displays was associated with their language skills.

## 2. Methods

### 2.1. Participants and Settings

A total of 23 children with autism (19 males; 4 females) were recruited to participate in this study through flyers distributed to existing research projects and preschools in North and South Carolina. Inclusion criteria were (a) an age between 3 years and 5 years, 11 months, (b) a clinical diagnosis of autism, and (c) meeting criteria for autism on the* Autism Diagnostic Observation Schedule *(*ADOS*) [35] previously administered by public school system psychologists or other research groups. All children displayed symptoms consistent with an autism diagnosis during their participation in this study, based on informal observation. Children were excluded if they had concomitant genetic diagnoses (fragile X syndrome, tuberous sclerosis, etc.). Vision and hearing were required to be within normal limits or to be corrected to within normal limits, as confirmed by record review and/or parent report.

Participants were seen during a single visit lasting less than one hour. The testing environments were child-friendly settings that were quiet and away from distraction of other activities, either in a lab or a separate room in the child's preschool.

### 2.2. Measures

To gauge the overall severity of symptoms of social relatedness in autism, the* Social Responsiveness Scale-Preschool for Three-Year-Olds *(*SRS-P*) [36], a validated 65-item parent questionnaire, was completed by each child's caregiver. The SRS-P is based upon the original version, the* Social Responsiveness Scale* (SRS) [37], and is only slightly different from the original version, with changes in wording of some items to make them more appropriate for younger children. Due to these minimal differences, the SRS-P for 3-year-olds was used for the entire sample. The SRS uses a rating scale from zero to three for each item. A score of 60 or greater is associated with an autism spectrum disorder [38].

The Auditory Comprehension portion of the* Preschool Language Scale-4 *(*PLS-4*) [39] was administered to measure receptive language. The* PLS-4* is a standardized test of expressive and receptive language skills designed for use with children from birth to 6 years, 11 months.

### 2.3. Design and Apparatus

We used a two-choice intermodal preferential looking paradigm [40], with the two competing stimuli being displayed on separate video monitors. It was selected for the current study because preferential looking toward synchrony is thought to reflect intersensory matching (perceiving multisensory stimuli as a cohesive unit) and integration by requiring that the participant detect and discriminate the intersensory relationship and then select an explicit response [41]. Benefits of temporal synchrony can only be derived given those conditions.

Two 19-inch computer monitors were placed side by side with a six-inch gap between them. A Canon VIXIA HF R100 camcorder, used to record looking behaviors, was placed behind and above the two monitors, centered between them, and a speaker to broadcast the auditory stimuli was placed in between the monitors with the volume set at a comfortable listening level similar to conversation. Video clips were held on a Macintosh minicomputer and stored in i-Tunes.

### 2.4. Stimuli

Four 30-second video clips featured four different dolls, each paired with a different name and different play set. Four generic dolls (two males and two females) were selected in order to decrease the likelihood that children would already be familiar with the dolls. The dolls were called “Kiku,” “Pilou,” “Nuwa,” and “Barra,” names selected because they were not likely to be familiar to the child, and their bisyllabic structure readily allowed for movement of the dolls in synchrony with speaking the dolls' names.

The midportion of the investigator's trunk was recorded as she held each doll and vertically bounced the doll in synchrony with the doll's name each time she uttered the name. Each video clip segment contained five statements about the play activity and included the doll's name once per statement. Therefore, each doll's name was presented a total of five times during each segment. The name of the doll was uttered with movement of the doll in synchrony with the double syllables resulting in a “double bounce.” The bounce was always done vertically and spanned approximately 6 inches. For example, the investigator said, “Kiku likes milk” while moving the doll during the production of “Kiku” and then demonstrating the doll drinking milk. Deliberate movement paired with auditory stimuli only occurred on screen during production of the doll's name. The doll drinking milk occurred after the statement was complete. The doll's name paired with movement was evident in only the synchronous version. The name of the doll came at the beginning of the sentence in 14 of 16 opportunities. The researcher inserted a slight pause after the name of the doll was uttered, which allowed the movement in the asynchronous video to occur before the initiation of the rest of the utterances. For 2 of the 16 utterances, the name of the doll was embedded in the utterance: “Let's put lotion on Kiku's feet” and “Let's clean Nuwa's booboo.” In the asynchronous version, movement occurred during the production of the word “feet” but did not follow the synchronous two-syllable movement pattern. The word “booboo” was produced with movement of the doll in the asynchronous video. This unintended synchrony was not felt to invalidate the segment because adults viewing the segment were immediately able to tell which condition was synchronized, as was the case in a similar design by Bahrick [42].

The investigator held the doll and materials at chest level and her face was not captured on tape. This prevented confounds of sound and lip movement synchrony, as the target linguistic event was the pairing of the referent (doll) with the speech cue (name) and not the mouth with the speech cue. In addition, this intentional avoidance of the investigator's face removed the need for face processing, a known deficit for children with autism (e.g., [43]). Each video monitor featured identical recordings, but on one monitor, the video was delayed by 700 milliseconds in order to provide the same auditory and visual stimuli with only temporal synchrony being manipulated. A 700-millisecond delay was selected because typically developing infants are able to identify nonspeech auditory and visual stimuli as asynchronous at 350 milliseconds [44] and speech stimuli at 633 milliseconds [45]. Stimuli presented with gaps greater than 700 milliseconds were considered independent events rather than asynchronous in Gogate et al. [34]. At the neurophysiological level, temporal binding of synchronous multisensory stimuli is essential for the perception of the stimuli as a single event and may be disrupted in autism [46]. According to findings in school-age children and adolescents with autism, temporal-binding windows are framed within ±300 milliseconds [47], and young children with autism might have even larger temporal-binding windows because the temporal-binding window is larger in infancy and gradually decreases with age in typical development [48]. Therefore, based on prior research from several sources, the 700-millisecond delay was assumed to allow for adequate detection of asynchronies among this sample.

### 2.5. Procedure

Each child was centered between the two monitors, at a comfortable viewing distance (25 inches away) with the monitors at eye-level. A brief intermission (30 seconds) occurred between each of the four 30-second video segments. Attention was cued between the monitors to serve as a fixation point prior to the commencement of each of the four segments. For each trial, the sound-synchronous version of the video segment played on one monitor while the sound-asynchronous version of the same video segment played on the other monitor. Counterbalancing occurred with order of doll presentation and side of synchrony (i.e., two segments were created for each doll—one with synchrony on the right and one with synchrony on the left). Each condition (synchronous and asynchronous) was presented twice on each side.

Occasionally, a child required cues to remain in his or her seat. This was accomplished by gently physically cuing him or her around the waist. No incidents of extreme fussiness occurred. No child was ever coaxed to look at one or another monitor.

### 2.6. Data Collection Methods and Coding

We used the first 15 seconds of each video segment for coding in order to be more consistent with existing methods on preferential looking paradigms (e.g., [18, 29]). The attention of infants during intermodal preferential looking tasks tends to be distributed in an increasingly random manner as time progresses in a trial [49, 50], so the first fifteen seconds of each trial were coded to mitigate effects of decreasing participation in the task and to obtain the most reliable measure. Using digital video manipulation, the first frame of each 500-millisecond segment was captured, yielding two freeze-frames per second. Trained research assistants coded the frames as directed toward (a) right monitor, (b) left monitor, and (c) neither screen. This yielded a frequency count for each type of looking direction for each 15-second segment. Although a saccade (rapid simultaneous movement of both eyes) can be as fast as 300 milliseconds in typically developing preschoolers [51], coding 500-millisecond frames was sufficient because there were no examples of rapid saccades back and forth between screens that would potentially change the results; that is, participants tended to look at a screen for several seconds before shifting attention either off screen or to the other screen. In addition, data from five randomly selected participants, each with four 15-second video recordings, were coded using a frame-by-frame (30 frames per second) method. This resulted in Pearson correlation of .991, *P* = .001, with the data coded for 500-millisecond frames. Coding fidelity was checked on 4 of the 23 participants by the first author yielding 97% agreement. Coders were blind to the stimulus conditions (synchronous or asynchronous) on each monitor. The four segments for each participant were matched to condition (synchronous/asynchronous) after all coding was completed. The PLS-4 was administered to examine language skills. The receptive language age-equivalent (A-E) scores were used to compute a receptive language ratio score (receptive language A-E/child's chronological age) rather than using a standard score due to 8 of the 23 children having standard scores at the floor for the PLS-4 (i.e., a score of 50 or below).  Table 1 summarizes the characteristics of the sample.

## 3. Results

### 3.1. Synchrony Detection

**Figure 1:**
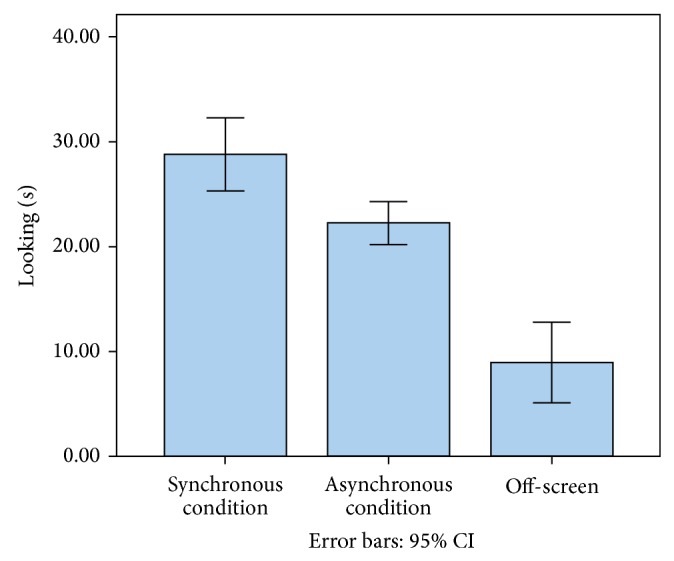
Preferential looking by stimulus condition.

Preferential looking to (1) a synchronous display condition and (2) an asynchronous display condition and (3) off-screen was evaluated in twenty-three preschool children with autism. Out of a total of 60 seconds of available looking time to synchrony and to asynchrony or off-screen, synchronous looking time ranged from a total of 15 to 40 seconds with a mean of 28.8 seconds (SD = 8.08). Asynchronous looking time ranged from a total of 15 to 31.50 seconds with a mean of 22.24 seconds (SD = 4.65). Off-screen looking time ranged from a total of .50 to 29.50 seconds with a mean of 8.93 (SD = 8.84) (see Figure 1). There were no significant side preferences *t*(22) = 1.37, *P* > .05, and no significant difference between time spent looking to synchrony for the first 7.5 seconds compared to the last 7.5 seconds *t*(22) = 1.54, *P* > .05. In addition, the condition to which the child first looked (4 opportunities per child) was coded to ensure that children were not simply directing attention to the object that moved first. Thirty-nine of 92 opportunities were first to the synchronous display (where the object moved first), 32 were to the asynchronous display, and 21 were off screen; thus, less than half of the first looks were to the synchronous display.

We used a percent of looking time to the synchronous display based on the first 15 seconds of recorded looking behavior (i.e., synchronous looking/[synchronous + asynchronous looking]; not including off-screen looking). Our sample looked to synchrony 55.75% of the time (see above for mean and standard deviations in seconds) and to asynchrony 44.25% of the time, as a proportion of total looking time to one screen or the other. Based upon a one-sample *t*-test against chance looking (50%), looking to synchrony occurred significantly longer than chance, *t*(22) = 3.14, *P* = .005. The percent of total looking to synchrony is the proportional inverse of total looking to asynchrony; therefore, the significant difference in looking to synchrony reflects the same significant difference in looking to asynchrony.

### 3.2. Associations with Language

**Figure 2:**
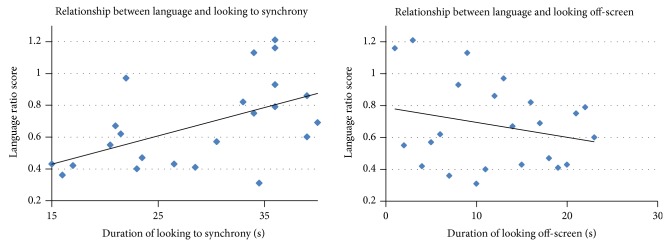
Language as a function of looking behavior.

A Pearson correlation revealed a strong, positive association between time looking to synchrony and language ratio scores, *r*(23) = .54, *P* = .008. Additionally, a Pearson correlation showed a negative association between off-screen looking and receptive language score, *r*(23) = .54, *P* = .007. See Figure 2.

## 4. Discussion

Twenty-three preschool children with autism were evaluated to determine (1) their ability to detect audio-visual temporal synchrony of objects paired with linguistic stimuli through a preferential looking paradigm and (2) the extent to which preferential looking to synchrony was associated with language skills. Our sample demonstrated preferential looking toward the visual presentation that was synchronous to the sound source as opposed to the visual display that was slightly out of synchrony with the sound source. An important methodological difference between the previous studies that found children with autism did not demonstrate preferential looking to synchrony using linguistic auditory stimuli and the present study is that the visual stimuli in the present study did not include faces but rather a person moving objects in synchrony with linguistic stimuli. This difference in findings could reflect difficulty with face processing or an overarching sensory processing deficit. The fine-grained movements associated with lip postures for speech may not have been salient enough to capture attention but the larger and simpler movement of an object bouncing could be more easily processed. Future studies should assess differences between synchrony detection with and without faces present as well as with high and low visual complexity in the same sample to determine the extent to which faces potentially interrupt audio-visual sensory processing in children with autism. If complexity does not impact synchrony detection like the presence of faces, it may be that attention is not being appropriately trained to prioritize the highly social nature of face stimuli, thus diminishing social motivation (for a review of the social motivation theory of autism, see Chevallier et al., 2012 [52]). Similarly, the question of whether or not synchronized object-voice stimulation is powerful enough to draw attention to objects being labeled, as is the case with typically developing children [28], requires further study.

Our sample demonstrated that more time spent looking to the temporally synchronized object-speech stimuli was significantly associated with higher language skills. Although our study does not determine causality, the association is in line with previous studies on typically developing infants and toddlers that show synchrony detection is associated with better attention to language and better language learning [16–20]. It is likely that development of synchrony detection precedes language development and guides attention to the most important aspects of communication. We might conclude that children with ASD who detect synchrony better are at an advantage in directing attention to linguistic content and are less likely to succumb to the ambiguity inherent in attempting to connect two unrelated stimuli (words with referents). As mentioned earlier, Gogate and colleagues suggested that delays in language development in preterm infants might be a result of early attenuations in synchrony detection [22]. Future studies should investigate a variety of populations with known delays in language development to elucidate potential underlying mechanisms of early lexical acquisition based on intersensory processing in various contexts. In addition, specific areas of language (e.g., phonology and pragmatics) should be teased out to determine if they are differentially associated with poorer intersensory processing.

The more time children spent looking off-screen, the worse their language skills became. Again, the direction of causality is unclear; it is possible that children with poorer language skills have less interest in looking to language-based video vignettes or that children with less interest in language-based stimuli learn language more slowly.

## 5. Conclusions

These findings demonstrate that children with autism can discriminate audio-visual temporal synchrony in certain contexts and may suggest that exaggerated multisensory stimuli aid in synchrony detection for these children. It should be noted that our experimental task purposefully obscured the actors face in the videos which may have improved attention to the multimodal stimuli; however, the findings underscore the potential clinical benefits of using exaggerated multisensory cues (i.e., moving objects in synchrony with auditory stimuli). Harnessing the benefits of intersensory redundancy may support better joint attention, language learning, and memory [53].

The correlational nature of this study precludes causal interpretations; thus, future longitudinal studies are needed to determine the extent to which synchrony detection impacts later language skills. Moreover, measures of intersensory processing may have diagnostic utility when applied to samples with infants at risk for ASD.

## Figures and Tables

**Table 1:** Sample characteristics.

Participant characteristics	Mean (SD)	Range
Chronological age (mos)	55.45 (7.54)	42–69
PLS-4 receptive language A-E	38.05 (15.30)	17–75
Receptive language ratio^*^	.69 (.06)	.31–1.21
Social responsiveness scale-P	83.32 (29.56)	19–122

^*^Receptive language ratio = PLS-4 receptive language A-E/chronological age.

## References

[1] American Psychiatric Association (2013). *Diagnostic and Statistical Manual of Mental Disorders*.

[2] Bahrick L. E., Lickliter R., Kail R. (2002). Intersensory redundancy guides early perceptual and cognitive development. *Advances in Child Development and Behavior*.

[3] Bahrick L. E., Lickliter R., Bremner A., Lewkowicz D. J., Spence C. (2012). The role of intersensory redundancy in early perceptual, cognitive, and social development. *Multisensory Development*.

[4] Patterson M. L., Werker J. F. (2003). Two-month-old infants match phonetic information in lips and voice. *Developmental Science*.

[5] Ratner N. B. (2013). Why talk with children matters: clinical implications of infant- and child-directed speech research. *Seminars in Speech and Language*.

[6] Smith N. A., Strader H. L. (2014). Infant-directed visual prosody: Mothers’ head movements and speech acoustics. *Interaction Studies: Social Behaviour and Communication in Biological and Artificial Systems*.

[7] Paul R., Chawarska K., Fowler C., Cicchetti D., Volkmar F. (2007). “Listen my children and you shall hear”: auditory preferences in toddlers with autism spectrum disorders. *Journal of Speech, Language, and Hearing Research*.

[8] Baron-Cohen S., Baldwin D. A., Crowson M. (1997). Do children with autism use the speaker's direction of gaze strategy to crack the code of language?. *Child Development*.

[9] Gogate L. J., Hollich G. (2010). Invariance detection within an interactive system: a perceptual gateway to language development. *Psychological Review*.

[10] Gibson E. J. (1969). *Principles of Perceptual Learning and Development*.

[11] Gogate L. J., Bahrick L. E., Watson J. D. (2000). A study of multimodal motherese: the role of temporal synchrony between verbal labels and gestures. *Child Development*.

[12] Brand R. J., Shallcross W. L. (2008). Infants prefer motionese to adult-directed action. *Developmental Science*.

[13] Brand R. J., Baldwin D. A., Ashburn L. A. (2002). Evidence for “motionese”: modifications in mothers' infant-directed action. *Developmental Science*.

[14] Koterba E. A., Iverson J. M. (2009). Investigating motionese: the effect of infant-directed action on infants' attention and object exploration. *Infant Behavior and Development*.

[15] Mundy P., Gomes A. (1998). Individual differences in joint attention skill development in the second year. *Infant Behavior and Development*.

[16] Gogate L. J. (2010). Learning of syllable-object relations by preverbal infants: the role of temporal synchrony and syllable distinctiveness. *Journal of Experimental Child Psychology*.

[17] Gogate L. J., Bahrick L. E. (1998). Intersensory redundancy facilitates learning of arbitrary relations between vowel sounds and objects in seven-month-old infants. *Journal of Experimental Child Psychology*.

[18] Gogate L. J., Bahrick L. E. (2001). Intersensory redundancy and 7-month-old infants' memory for arbitrary syllable-object relations. *Infancy*.

[19] Gogate L. J., Prince C. G., Matatyaho D. J. (2009). Two-month-old infants' sensitivity to changes in arbitrary syllable-object pairings: the role of temporal synchrony. *Journal of Experimental Psychology: Human Perception and Performance*.

[20] Matatyaho D. J., Gogate L. J. (2008). Type of maternal object motion during synchronous naming predicts preverbal infants' learning of word-object relations. *Infancy*.

[21] Rader N. D. V., Goldring-Zukow P. (2010). How the hands control attention during early word learning. *Gesture*.

[22] Gogate L., Maganti M., Perenyi A. (2014). Preterm and term infants' perception of temporally coordinated syllable-object pairings: implications for lexical development. *Journal of Speech, Language, and Hearing Research*.

[23] Iarocci G., McDonald J. (2006). Sensory integration and the perceptual experience of persons with autism. *Journal of Autism and Developmental Disorders*.

[24] Marco E. J., Hinkley L., Hill S. S., Nagarajan S. S. (2011). Sensory processing in autism: a review of neurophysiologic findings. *Pediatric Research*.

[25] Bahrick L. E., Todd J. T., Stein B. E. (2012). Multisensory processing in autism spectrum disorders: Intersensory processing disturbance as a basis for atypical development. *The New Handbook of Multisensory Processes*.

[26] Happé F., Frith U. (2006). The weak coherence account: detail-focused cognitive style in autism spectrum disorders. *Journal of Autism and Developmental Disorders*.

[27] Baron-Cohen S., Ashwin E., Ashwin C., Tavassoli T., Chakrabarti B. (2009). Talent in autism: hyper-systemizing, hyper-attention to detail and sensory hypersensitivity. *Philosophical Transactions of the Royal Society B: Biological Sciences*.

[28] Walker-Andrews A. S., Haviland J. M., Huffman L., Toci L. (1994). Brief report: Preferential looking in intermodal perception by children with autism. *Journal of Autism and Developmental Disorders*.

[29] Bebko J. M., Weiss J. A., Demark J. L., Gomez P. (2006). Discrimination of temporal synchrony in intermodal events by children with autism and children with developmental disabilities without autism. *Journal of Child Psychology and Psychiatry and Allied Disciplines*.

[30] Bahrick L. E., Todd J. T., Vaillant-Molina M., Sorondo B. M., Ronacher C. H. Impaired detection of temporal synchrony for social and nonsocial events in children with autism spectrum disorders.

[31] Dawson G., Webb S. J., McPartland J. (2005). Understanding the nature of face processing impairment in autism: insights from behavioral and electrophysiological studies. *Developmental Neuropsychology*.

[32] Webb S. J., Dawson G., Bernier R., Panagiotides H. (2006). ERP evidence of atypical face processing in young children with autism. *Journal of Autism and Developmental Disorders*.

[33] Yu C., Smith L. B. (2012). Embodied attention and word learning by toddlers. *Cognition*.

[34] Gogate L. J., Bolzani L. H., Betancourt E. A. (2006). Attention to maternal multimodal naming by 6- to 8-month-old infants and learning of word-object relations. *Infancy*.

[35] Lord C., Rutter M., Dilavore P., Risi S. (1999). *The Autism Diagnostic Observation Schedule (ADOS)*.

[36] Pine E., Luby J., Abbacchi A., Constantino J. N. (2006). Quantitative assessment of autistic symptomatology in preschoolers. *Autism*.

[37] Constantino J. N. (2002). *The Social Responsiveness Scale*.

[38] Constantino J. N., Davis S. A., Todd R. D. (2003). Validation of a brief quantitative measure of autistic traits: comparison of the social responsiveness scale with the Autism Diagnostic Interview-Revised. *Journal of Autism and Developmental Disorders*.

[39] Zimmerman I., Steiner V., Pond R. E. (2002). *Preschool Language Scale*.

[40] Spelke E. (1976). Infants' intermodal perception of events. *Cognitive Psychology*.

[41] Lewkowicz D. J. (2000). The development of intersensory temporal perception: an epigenetic systems/limitations view. *Psychological Bulletin*.

[42] Bahrick L. E. (1983). Infants' perception of substance and temporal synchrony in multimodal events. *Infant Behavior and Development*.

[43] Monk C. S., Weng S.-J., Wiggins J. L. (2010). Neural circuitry of emotional face processing in autism spectrum disorders. *Journal of Psychiatry and Neuroscience*.

[44] Lewkowicz D. J. (1996). Perception of auditory-visual temporal synchrony in human infants. *Journal of Experimental Psychology: Human Perception and Performance*.

[45] Lewkowicz D. J. (2003). Learning and discrimination of audiovisual events in human infants: the hierarchical relation between intersensory temporal synchrony and rhythmic pattern cues. *Developmental Psychology*.

[46] Brock J., Brown C. C., Boucher J., Rippon G. (2002). The temporal binding deficit hypothesis of autism. *Development and Psychopathology*.

[47] Foss-Feig J. H., Kwakye L. D., Cascio C. J. (2010). An extended multisensory temporal binding window in autism spectrum disorders. *Experimental Brain Research*.

[48] Lewkowicz D. J. (2010). Infant perception of audio-visual speech synchrony. *Developmental Psychology*.

[49] MacRoy-Higgins M., Schwartz R. G., Shafer V. L., Marton K. (2013). Influence of phonotactic probability/neighbourhood density on lexical learning in late talkers. *International Journal of Language and Communication Disorders*.

[50] Schafer G., Plunkett K. (1998). Rapid word learning by fifteen-month-olds under tightly controlled conditions. *Child Development*.

[51] Fukushima J., Hatta T., Fukushima K. (2000). Development of voluntary control of saccadic eye movements: I. Age- related changes in normal children. *Brain and Development*.

[52] Chevallier C., Kohls G., Troiani V., Brodkin E. S., Schultz R. T. (2012). The social motivation theory of autism. *Trends in Cognitive Sciences*.

[53] Leekam S. R., Hunnisett E., Moore C. (1998). Targets and cues: gaze-following in children with autism. *Journal of Child Psychology and Psychiatry*.

